# First successful case of in vitro fertilization-embryo transfer with venom immunotherapy for hymenoptera sting allergy

**DOI:** 10.1186/1476-7961-2-11

**Published:** 2004-10-19

**Authors:** Eric Scott Sills, Susan C Conway, Carolyn R Kaplan, Mark Perloe, Michael J Tucker

**Affiliations:** 1 Georgia Reproductive Specialists, Division of Reproductive Endocrinology and Infertility, Department of Obstetrics and Gynecology, Atlanta Medical Center; Atlanta, Georgia USA

**Keywords:** allergy, hymenoptera, IgE, immunology, in vitro fertilization

## Abstract

**Background:**

To describe immune and endocrine responses in severe hymenoptera hypersensitivity requiring venom immunotherapy (VIT) during in vitro fertilization (IVF).

**Case presentation:**

A 39-year old patient was referred for history of multiple miscarriage and a history of insect sting allergy. Four years earlier, she began subcutaneous injection of 100 mcg mixed vespid hymenoptera venom/venom protein every 5–6 weeks. The patient had one livebirth and three first trimester miscarriages. Allergy treatment was maintained for all pregnancies ending in miscarriage, although allergy therapy was discontinued for the pregnancy that resulted in delivery. At our institution ovulation induction incorporated venom immunotherapy (VIT) during IVF, with a reduced VIT dose when pregnancy was first identified. Serum IgE was monitored with estradiol during ovulation induction and early pregnancy. Response to controlled ovarian hyperstimulation was favorable while VIT was continued, with retrieval of 12 oocytes. Serum RAST (yellow jacket) IgE levels fluctuated in a nonlinear fashion (range 36–54%) during gonadotropin therapy and declined after hCG administration. A healthy female infant was delivered at 35 weeks gestation. The patient experienced no untoward effects from any medications during therapy.

**Conclusion:**

Our case confirms the safety of VIT in pregnancy, and demonstrates RAST IgE can remain <60% during IVF. With proper monitoring, VIT during IVF can be safe and appropriate for selected patients and does not appear to adversely affect blastocyst implantation, early embryo development or perinatal outcome. Further studies will be needed to develop VIT guidelines specifically applicable to IVF.

## Introduction

Insect sting allergies affect approximately 3% of the general population, and patients with insect sting allergy during pregnancy are generally advised to continue venom immunotherapy (VIT). However, there have been no descriptions of VIT during infertility therapy despite increased utilization of the advanced reproductive technologies [1]. In this report, we present endocrine and immunologic parameters observed during a successful in vitro fertilization cycle where a standard insect sting allergy protocol was used.

## Case report

A 39 year-old Caucasian G_4_P_1031 _was referred for evaluation and management of recurrent pregnancy loss. Medical history was significant for known carrier state for β-thalassemia. Mild hypothyroidism had been diagnosed in 2002 with immediate initiation of replacement therapy. The patient was a non-smoker, in good general health and had no gynecologic complaint. BMI was 21.7 kg/m^2^. In 1997, she experienced a severe hypotensive anaphylactic reaction following a yellow jacket sting (*Vespula *spp.) resulting in a full allergy work-up. The patient began subcutaneous injection of 100 mcg mixed vespid hymenoptera venom/venom protein (Pharmalgen^®^; ALK Abello, Hørsholm, Denmark) every 5–6 weeks, which was well tolerated.

All four conceptions were established without medical assistance, involved the same partner, and were achieved after the hymenoptera hypersensitivity diagnosis. The initial pregnancy occurred three years before presentation and resulted in a first trimester spontaneous abortion. No adjustment was made to the allergy injection regimen during that pregnancy. Fetal cardiac activity was initially present, but was lost at 10 weeks' gestation for unknown reasons. No curettage was performed.

One year later, a second pregnancy was established but for this pregnancy hymenoptera venom therapy was discontinued when pregnancy was first recognized (~6 weeks). A 3170 g female infant was delivered vaginally at 40 1/2 weeks' gestation. In 2001 and 2002, the patient established two additional pregnancies and hymenoptera therapy was maintained at 5–6 week intervals for both; both resulted in first trimester spontaneous abortions. For these miscarriages, dilation and curettage was undertaken but no karyotype was performed and no cause for the losses was identified.

At our institution, euthyroid status was verified, the thalassemia carrier state was confirmed, and we identified a new homozygous A223V mutation at the methyltetrahydrofolate reductase (MTHFR) locus. Folic acid intake was immediately increased to 800 mcg/d, although a baseline serum homocysteine level was not measured. Factor V Leiden, protein S, protein C, and other coagulation tests were normal, as were karyotypes obtained from both partners. Anticardiolipin, antiphospholipid and antiovarian antibody titres were all negative. However, transvaginal saline uterine sonography revealed a uniform 5 mm echodense lesion consistent with an endometrial polyp. Outpatient hysteroscopic polypectomy was performed without complication. After discussing various infertility therapies and associated success rates given her age, the patient elected to undergo IVF.

**Figure 1:**
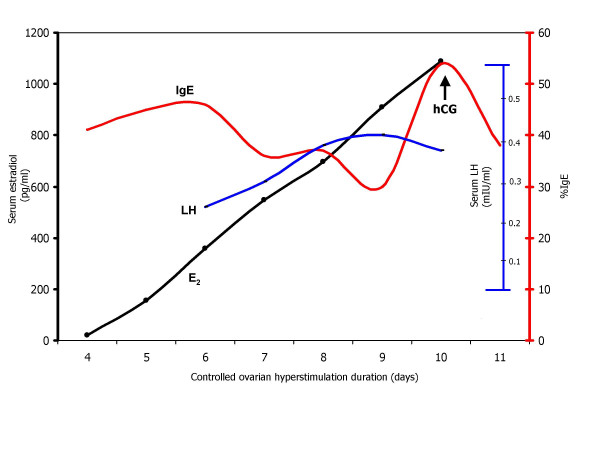
Relationships among serum luteinizing hormone (LH-blue), estradiol (E2-black), and yellow jacket RAST %IgE (IgE-red) observed during in vitro fertilization and concomitant venom immunotherapy.

In March 2003, the patient began programmed ovarian hyperstimulation using a combined recombinant-FSH+hMG protocol (300 IU/d Humegon^®^, Ferring Pharmaceuticals Inc.; Tarrytown, NY USA and 300 IU/d Gonal-F^®^, Serono Labs; Norwell, MA USA). Pre-treatment pituitary downregulation was achieved via 5 u/d leuprolide acetate and was continued × 3 d after gonadotropin therapy commenced. No alteration was made in the patient's allergy injection sequence during ovulation induction (*i.e*., 100 mcg every 5–6 weeks), and serum yellow jacket RAST IgE measurements were obtained via commercial fluoroimmunoassay including positive and negative controls (UniCAP^® ^IgE kit, Pharmacia Diagnostics, Uppsala, Sweden). While absolute IgE levels remained <0.35 kU/l throughout therapy, percentage IgE results were variable and these data are summarized in Figure 1.

On cycle day 10, subcutaneous hCG (10,000 IU) was given [2] with serum estradiol at 1090 pg/ml. Twelve oocytes were retrieved and 7 advanced to the 2*pn *stage following conventional insemination. A four-day course of methylprednisolone (16 mg/d) was started on the day of oocyte retrieval. On post-fertilization day three, the ultrasound-guided transfer of four embryos was performed. Immediately following embryo transfer, the patient was placed on oral aspirin (81 mg/d) and subcutaneous heparin (5,000 IU b.i.d). Luteal phase support was administered as daily 50 mg IM progsterone in oil injections.

Two weeks after embryo transfer, serum hCG was 72 mIU/ml. On May 5, 2003, transvaginal ultrasound confirmed a single intrauterine pregnancy with fetal cardiac rate at 126/min. Progesterone was discontinued at the 10^th ^gestational week. Immunology and perinatology consultants agreed with reduced dose allergy protocol through the third trimester, and hymenoptera venom protein extract (75 mcg) treatment was maintained to 32 weeks gestation. The patient experienced no untoward reaction or hypersensitivity to gonadotropins, VIT, or supplementary progesterone during therapy.

At 32 weeks, obstetrical sonogram suggested reduced amniotic fluid levels and the patient was given intramuscular betamethasone (12 mg/d × 2 days) and placed on bedrest. At this point allergy injections were discontinued since the patient was not outdoors and risk for insect sting was regarded as low. Intravenous oxytocin was started at 35 weeks due to oligohydramnios and resulted in vaginal delivery of a 2495 g female infant. Mother and baby were discharged home after an uncomplicated two-day postpartum course. Allergy injections resumed (100 mcg every 5–6 weeks) when breastfeeding was completed three months later.

## Conclusion

Overall, the incidence of allergy to insect stings is ~3% in adults [3], with allergy to hymenoptera species venom comprising an important subset of this population. Whether or not severe insect sting allergy contributes to poor reproductive outcome has been discussed in earlier reports [3-5], yet the immunology of pregnancy remains complex and poorly understood. While connections between infertility/spontaneous abortion and immune dysfunction have been explored by others [6,7], the reported conclusions have been highly variable [8,9]. In addition, difficulty with immunoassay standardization has made some findings difficult to reproduce [10].

Although continuation of allergy therapy during pregnancy is generally recommended [11], the interaction between ovulation induction agents and hymenoptera venom therapy has never been characterized. Our patient experienced no hypersensitivity or untoward effects during allergy therapy and gonadotropin use; both were well tolerated when administered together. We observed a variable IgE pattern, with a gradually increasing IgE response progressing with follicular growth during ovulation induction. Interestingly, a sharply diminished IgE level was registered immediately after hCG administration. The significance of the reduced terminal immunoglobulin titre is unkown but may reflect an immunomodulatory attenuation effect of hCG and/or progesterone [12].

Evaluation of this patient with a history of multiple spontaneous abortions identified additional factors which might contribute to a poor reproductive outcome. Specifically, endometrial polyps [13] and homozygous MTHFR mutation [14] are recognized independent risk factors for miscarriage. In addition to venom protein, our patient received other medications which modify immune response including aspirin [15], heparin [16], progesterone [17], and human chorionic gonadotropin [12]. Methylprednisolone [18] was administered at embryo transfer and betamethasone was given near delivery [19]. While for our patient VIT was a component of therapy culminating in a satisfactory reproductive outcome, the result should be recognized as the sum of all clinical interventions and insufficient data exists to ascribe specific roles for individual treatments. Levels of IgG4 blocking antibody were not quantified in this study, although measurement of this parameter in subsequent studies may offer additional insight into potential mechanisms of protection (*i.e*., reduction of miscarriage risk).

It has been hypothesized that early production of IL-10 associated with VIT may induce T-cell anergy, dampening T helper type 2 response and resulting in a T helper type 1 dominant cytokine response. As the role of T helper 1 type immune response in blastocyst implantation becomes more completely characterized, T helper type 1 function may prove to be important in early placental dysfunction or recurrent pregnancy loss [20]. These potential mechanisms notwithstanding, when anatomic and hematologic abnormalities were corrected and VIT for insect sting allergy therapy was continued, a healthy livebirth after IVF was achieved. Our report offers, for the first time, reassurance for women undergoing IVF who also suffer from severe insect sting allergy requiring VIT. Data derived from further studies will be helpful as VIT guidelines are developed specifically for patients undergoing IVF.

## Competing interests

The authors declare that they have no competing interests.

## Authors' contributions

ESS was the principal physician and coordinated the research. SCC, CRK and MP edited the manuscript. MJT was chief embryologist and edited the manuscript.
